# Understanding of front of package nutrition labels: Guideline daily amount and warning labels in Mexicans with non-communicable diseases

**DOI:** 10.1371/journal.pone.0269892

**Published:** 2022-06-24

**Authors:** Janine Sagaceta-Mejía, Lizbeth Tolentino-Mayo, Carlos Cruz-Casarrubias, Claudia Nieto, Simón Barquera

**Affiliations:** Center for Health and Nutrition Research, National Institute of Public Health, Cuernavaca, Mexico; Universidad Autónoma de Coahuila, MEXICO

## Abstract

One strategy for the prevention and treatment of non-communicable diseases (NCDs) is the implementation of the front-of-pack labeling (FoPL) in foods and beverages. In 2020, Mexico adopted the warning label system (WL) as a new public health policy, whose aim is to help consumers make healthier food choices. Previously, the Guideline Daily Amount (GDA) was the labelling used it. This paper aims to compare the understanding of two FoPL, the GDA and the WL, through the identification of unhealthy products in Mexicans with NCDs. We analyzed data from 14,880 Mexican adults older than 20 years old with NCDs (overweight-obesity (*OW/O*), self-reported diabetes mellitus 2 (*DM2*), or/and hypertension (*HT*), or/and dyslipidemia (*Dys*)). Participants were randomly assigned to one of two groups: the GDA labeling or WL. Each group had to respond to a survey and had to classify food products images as healthy or unhealthy according to the labelling system to which they were assigned. The correct classification was determined according to the criteria of Chile’s labeling nutrient profile stage 3. To evaluate the correct classification in each one of the groups we evaluated the differences in proportions. Logistic regression models were used to assess the likelihood to correctly classify the product according to participants’ number of diseases and WL information, taking GDA label as a reference. Participants who used the information contained in the GDA label misclassified food product labels in greater proportion (70%), mostly participants with three or more NCDs (participants with *OW/O+ HT+ Dys*, represent 42.3% of this group); compared with those who used WL (50%). The odds of correct classification of food products using WL image were two times greater compared to GDA image in participants with NCDs; being greater in participants with *three or more NCDs*. The study results highlight the usefulness of WL as it helps Mexicans with NCDs to classify unhealthy food products more adequately compared with GDA.

## Introduction

Overweight, obesity, and other non-communicable diseases (NCDs) are priority public health problems in Mexico that can be prevented with changes in the food environment [1, 2]. In Mexico, the prevalence of adults with overweight and obesity (*OW/O*) is 75.2% [3], dyslipidemia (*Dys*) 19.5%, hypertension (*HT*) 18.4%, and diabetes mellitus 2 (*DM2*) 10.3% [4]. However, the prevalence of NCDs is higher for adults under vulnerable conditions (i.e., Mexicans with elementary school level education or less, living in rural localities, and low socioeconomic status) [5]. In Mexico, cardiovascular diseases and *DM2* were the leading causes of health-related mortality in 2020 [6].

Given the high rates of morbidity and mortality attributable to diet-related NCDs, health authorities adopted public health policies to promote healthier diets [7]. The World Health Organization (WHO) recommends the implementation of front-of-pack labelling (FoPL) on less healthy foods and beverages as a strategy to control and prevent NCDs [8]. Different types of FoPL have been developed based on local evidence about acceptability, comprehension, and effect on decision making at the point of sale [9, 10]. Some of them highlight if the food product is healthy based on certain criteria (e.g., Health Rating Stars, Keyhole and Choices), others warn if the food products have excess of nutrients of concern (e.g., Warning Labelling and Multiple Traffic Light) and others, like the GDA, indicate the contribution of nutrients to the diet [11].

FoPL aims to provide nutritional information of products to help consumers make healthier food choices [12, 13], and decrease their mortality risk caused from NCDs by reducing the intake of critical nutrients and promoting healthier diets [7, 14]. Critical nutrients such as sodium, free sugar, saturated fat and trans fat are the main components linked to the development of NCDs when their consumption is excessive [8, 9, 15], and are frequently found in processed and ultra-processed products (UPP) (formulations of ingredients, most of them for exclusive industrial use and involves several procedures and industries, characterized for containing excessive amounts of critical nutrients and other food additives such as colorants, flavorings, texturizers, or sweeteners, also known as "cosmetic additives", to make them attractive to sight, taste, smell, and therefore make them hyperpalatable) [11, 16]. According to the Pan-American Health Organization (PAHO), Mexico was the second highest country in sales of UPP in Latin America during the period 2009–2014 [12]. The intake of UPP in the Mexicans’ diet represents approximately 20–30% of total calories and has been associated with a lower dietary quality which is characterized by a lower intake of protein and dietary fiber [13, 17].

In October 2020, Mexico adopted the warning label system (WL) as a public health policy in foods and non-alcoholic beverages (FAB), such was published in the Official Gazette (NOM-051, by its acronym in Spanish) [18]. This regulation is mandatory for FAB that exceed limits for energy, sodium, added sugar, saturated fats, and trans fats [19]. Before this regulation, from 2014 to 2020, the GDA was the FoPL implemented as part of a mandatory public health policy as response to the Ministry of Health to address the increase in *OW/O* and promote healthy dietary options, but was incorporated in 2011 as a voluntary initiative by the food industry [20, 21]. The GDA labelling present a variety of obstacles for its interpretation as it required nutrition knowledge and mathematical skills [22], making it difficult for consumers to determine the healthfulness of the food or beverage [23, 24]. In a recent study, less than a third of Mexicans were able to adequately identify an unhealthy product using the GDA label, indicating its understanding is low [25], especially by vulnerable population [24]. In contrast, there is evidence of the better understanding using WL [9, 10].

To date we did not find evidence about the understanding WLs in participants with NCDs; therefore, the aim of this paper is to compare the understanding between two FoPL, GDA vs WL, through the correct identification of unhealthy food products in Mexicans with *OW/O* and NCDs.

## Materials and methods

Sample design and selection: This study used data from the 2018 National Health and Nutrition Survey (ENSANUT by its acronym in Spanish), which has a cross-sectional, probabilistic, multistage, and stratified design. The methodology, including the description of the sample and logistic design, has been previously published [26]. Participants older than 20 years old and who were literate were asked to classify two food product labels with the information contained in them. Written consent was obtained from all participants and they could withdraw from the study at any time. All data was treated as confidential. The study protocol was approved by the Ethics, Research and Biosafety Committee of the National Institute of Public Health (INSP-Spanish acronym).

### Sociodemographic information

Sex, age, and education level were obtained for all participants. Age was categorized in decades (20–29, 30–39, 40–49, 50–59, and ≥60) and education level was classified as elementary school or less, middle school, and high school or more. Socioeconomic level was determined as low, medium-low, medium-high, and high according to the indicators defined in the survey design [27]. Locality was categorized according to the number of inhabitants as rural (<2,500 inhabitants) and urban (>2,500 inhabitants). Finally, the country was divided into 4 regions: South, North; Central; and Mexico City.

### Understanding of GDA and WL

To evaluate the understanding of two nutrition label systems used in Mexico (former GDA and current WL), two questionnaires were designed and randomly assigned to participants. Half of the survey participants received the GDA label questionnaire while the other half received the WL questionnaire.

**Fig 1:**
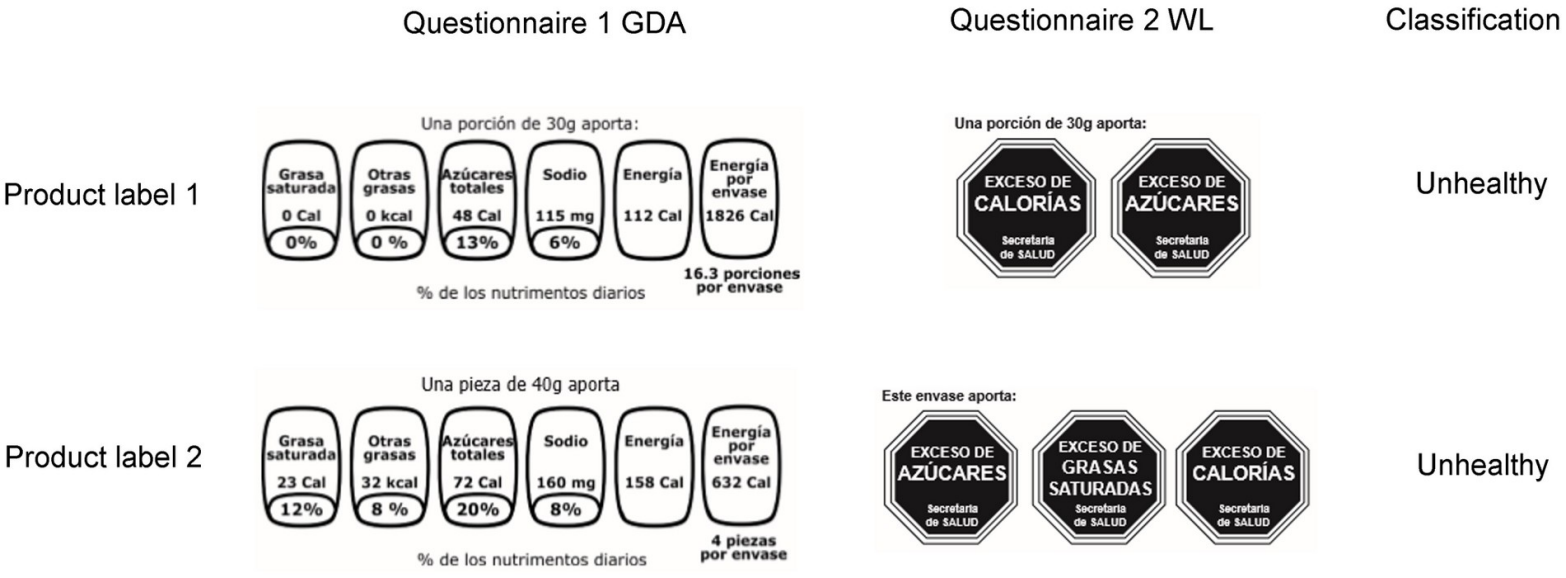
Images used in questionnaires one (GDA) and two (WL) of two product labels used for the assessment of understanding and their corresponding classification. GDA and WL images of two food products shown to the participants. *A food product is unhealthy when it contains excess of calories and/or critical nutrients. The reference cut-off points were based on Chile’s labelling nutrient profile stage 3 criteria.

The questionnaires had images of nutrition labels from the same two food products (Fig 1) and differed only by nutrition label format (GDA or WL). For each image, the question asked was: “Considering the information on the label, could you respond if the product is…” *very healthy*, *healthy*, *less healthy*, and *unhealthy”*.

### Correct classification

Food products were classified according to the FoPL type showed to participants, GDA or WL. The food products were considered as unhealthy according to Chile’s labelling nutrient profile stage 3 criteria [28]. The answers “*very healthy*, *healthy”* were considered as incorrect classification and the answer “*unhealthy”* was considered as correct.

### Self-reported non-communicable diseases (NCDs)

We considered that a participant had hypertension (*HT*), diabetes mellitus 2 (*DM2*) and dyslipidemia (*Dys*) when they self-reported based on an earlier medical diagnosis. Dyslipidemia is considered with self-reported hypertriglyceridemia and/or hypercholesterolemia. To determine the prevalence of previously diagnosed NCDs we used the affirmative response of the following questions: “Has a doctor ever told that you have diabetes or high blood sugar?”, “Has a doctor ever told that you have high blood pressure?”, “Has a doctor ever told that you have high cholesterol?”, “Has a doctor ever told that you have high triglycerides?”.

### Overweight and obesity

Body Mass Index (BMI) was calculated with the anthropometric measurements of height and weight. We used a BMI of ≥25Kg/m^2^ as the cut-off points used to determine overweight and obesity (*OW/O*) as recommended by the WHO.

### Number of non-communicable diseases

This variable was created with the number of self-reported NCDs and if the participant had *OW/O*; finding a range from 0 to 3 or more diseases, where 0 corresponds to the absence of NCDs and ≥3 is the maximum number of NCDs.

### Statistical analysis

All the variables included in this study were categorical. Frequencies, proportions and 95% confidence intervals were included. The Χ^2^ test was performed to examine differences between sample assigned to the GDA and WL questionnaire (Table 1).

**Table 1:** Sociodemographic characteristics of the population by type of food labeling.

	Total population	GDA	WL	P-value
	n = 14,880	n = 7,479	n = 7,401	
	% (95%CI)	% (95%CI)	% (95%CI)	
Total		50.5 (49.5;51.5)	49.5 (48.5;50.5)	
**Sex**				0.911
Women	57.5 (56.4,58.7)	57.1 (55.4,58.8)	57.2 (55.5,59.0)	
Men	42.5 (41.4,43.7)	42.9 (41.2,44.7)	42.8 (41.0,44.5)	
**Age (years)**				0.701
20–29	24.8 (23.6,26.0)	25.4 (23.8,27.0)	24.2 (22.7,25.8)	
30–39	20.9 (19.9,21.9)	21.0 (19.7,22.4)	20.7 (19.4,22.1)	
40–49	21.0 (20.1,22.0)	20.9 (19.5,22.3)	21.2 (19.8,22.6)	
50–59	15.95 15.0,16.9)	15.5 (14.2,16.8)	16.5 (15.2,17–8)	
≥60	17.4 (16.4,18.4)	17.3 (16.0,18.7)	17.5 (16.2,18.8)	
**Education level**				0.747
≤Elementary school	28.5 (27.3,29.7)	28.8 (27.3,30.3)	28.2 (26.6,29.8)	
Middle school	30.9 (29.7,32.2)	30.5 (28.9,32.2)	31.3 (29.7,32.3)	
≥High school	40.6 (39.2,42.0)	40.7 (38.9,42.5)	40.5 (38.6,42.4)	
**Region**				0.603
South	31.3 (28.9,33.8)	31.9 (29.4,34.6)	30.7 (28.1,33.4)	
North	19.5 (17.8,21.2)	19.2 (17.5,21.1)	19.7 (17.9,21.6)	
Center	35.8 (32.9,38.8)	35.3 (32.5,38.3)	36.2 (33.0,39.5)	
Mexico City	13.5 (11.7,15.5)	13.5 (11.6,15.8)	13.4 (11.3,15.9)	
**Locality**				0.440
Rural	25.6 (23.2,28.2)	25.9 (23.4,28.6)	25.3 (22.7,28.1)	
Urban	74.4 (71.8,76.8)	74.1 (71.4,76.6)	74.7 (71.9,77.3)	
**Socioeconomic level**				0.039
Low	19.8 (18.1,21.7)	20.2 (18.4,22.1)	19.3 (17.3,21.6)	
Middle-low	52.7 (50.6,54.9)	52.3 (50.1,54.6)	53.5 (50.8,56.1)	
Middle-high	20.1 (18.6,21.7)	19.4 (17.8,21.1)	21.0 (19.0,23.1)	
High	7.4 (6.5,8.4)	8.1 (6.9,9.4)	6.6 (5.7,7.8)	
**Number of diseases**				0.679
0	18.9 (17.9,19.9)	18.6 (17.4,19.9)	19.2 (17.9,20.5)	
1	48.7 (47.5,50.0)	49.1 (47.3,50.8)	48.3 (46.6,50.0)	
2	20.9 (19.9,21.9)	21.2 (19.8,22.7)	20.7 (19.3,22.1)	
≥3	11.5 (10.7,12.4)	11.2 (10.1,12.3)	11.5 (10.7,12.4)	

Abbreviations: 95% CI, 95% Confidence Interval, GDA: Guideline Daily Amount, WL: Warning Label

P-value obtained from the test Χ^2^

Tests of difference between two proportions were performed to evaluate the difference between the correct classification of the two food products using the GDA and WL images according to the number of diseases and NCDs. The results were stratified according to NCDs and number of diseases.

A logistic regression models were used to assess the likelihood of correctly classify two product labels according to WL in participants with different number of NCDs; the reference group was the GDA label. The models were adjusted by potential confounding variables including sex, age, education level, locality, area, region, and socioeconomic stratum. Analyses were performed adjusting for the complex design of the survey and a p-value <0.05 was used for statistical significance. Statistical analysis was performed in STATA V14.

## Results

We analyzed data from 14,880 adults older than 20 years old that represent 27,291,772 Mexican adults when expansion factors were applied; of which 57.5% (95% CI 56.4, 58.7) were women. We did not find any differences in socio-demographic characteristics nor in number of NCDs between participants who answered the GDA and those participants who answered the WL questionnaire; except for socioeconomic level (Table 1).

According to the number of diseases, most of the population had one (48.7% 95% CI:47.5,50.0) or two diseases (20.9% 95%CI:19.9,21.9), only 18.9% (95%CI: 17.9,19.9) had none. The most frequent NCDs were *OW/O* (91.4%, 95%CI: 90.3,92.5) for the one disease category, *OW/O+Dys* (47.3%, 95%CI 44.6,50.0) for the two diseases category and *OW/O +HT+Dys* (42.3%, 95%CI 38.5,46.1) for the three or more diseases category (Table 2).

**Table 2:** Prevalence of number and type of non-communicable diseases in Mexican population.

Condition	Percentage % (95% CI)
**Number of diseases** (n = 14,880; N expanded = 27,291,772)	
0	18.9 (17.9,19.9)
1	48.7 (47.5,50.0)
2	20.9 (19.9,21.9)
≥3	11.5 (10.7,12.4)
**Type of NCDs in participants with 1 disease** (n = 7,262; N expanded = 13,290,173)	
OW/O	91.4 (90.3,92.5)
HT	2.8 (2.3,3.4)
DM2	1.8 (1.4,2.3)
Dys	4.0 (3.1,4.9)
**Type of NCDs in participants with 2 diseases** (n = 3,137; N expanded = 5,708,654)	
OW/O+HT	34.8 (32.1,37.6)
OW/O+DM2	12.3 (10.7,14.0)
OW/O+Dys	47.3 (44.6,50.0)
HT+DM2	1.3 (0.9,2.1)
HT+Dys	2.4 (1.7,3.5)
DM2+Dys	1.9 (1.4,2.7)
**Type of NCDs in participants with 3 diseases** (n = 1,784; N expanded = 3,140,736)	
OW/O+HT+Dys	42.3 (38.5,46.1)
OW/O+HT+DM2	16.5 (14.1,19.1)
OW/O+DM2+Dys	18.2 (15.7,21.0)
HT+DM2+Dys	1.6 (0.9,2.6)
OW/O+HT+Dys+DM2	21.5 (18.8,24.4)

Abbreviations: 95% CI: 95% Confidence Interval, NCDs: Non-communicable diseases, OW/O: Overweight/Obesity; HT: Hypertension; DM2: Diabetes Mellitus 2; Dys: Dyslipidemia

The understanding evaluation of the GDA label and WL (Table 3), showed that participants who used the GDA label misclassified both food products in greater proportion compared to those participants who used the WL. The proportion of people with NCDs who correctly classified products labels decreased according to the number of diseases, regardless of the FoPL type used.

**Table 3:** Proportions of correct classification of two product labels according to the number of diseases and type of non-communicable diseases.

	Type of FoPL		P-value
	GDA	WL	
	% (95%CI)	% (95%CI)	
**Product label 1** *			
**Number of diseases**			
0	25.6 (21.5,30.1)	45.6 (40.6,50.6)	<0.001
1	27.2 (24.5,30.1)	51.9 (48.6,55.2)	<0.001
2	25.5 (21.7,29.6)	48.8 (44.3,53.3)	<0.001
≥3	22.6 (17.4,28.8)	46.3 (40.7,51.9)	<0.001
**Type of NCDs**			
OW/O	27.8 (24.5,30.8)	52.4 (49.0,55.7)	<0.001
OW/O+HT	26.9 (21.9,32.6)	49.7 (43.4,56.1)	<0.001
OW/O+HT+Dys	21.9 (14.3,32.0)	49.6 (40.3,58.9)	<0.001
**Product label 2** **			
**Number of diseases**			
0	36.2 (31.8,40.9)	51.9 (47.0,56.7)	<0.001
1	39.5 (36.6,42.6)	56.4 (53.1,59.7)	<0.001
2	33.0 (29.1,37.2)	54.0 (49.7,58.3)	<0.001
≥3	29.7 (24.6,35.3)	50.2 (44.7,55.7)	<0.001
**Type of NCDs**			
OW/O	41.1 (38.0,44.3)	57.2 (53.9,60.4)	<0.001
OW/O+HT	40.1 (34.3,46.2)	57.0 (50.8,62.9)	<0.001
OW/O+HT+Dys	27.3 (20.3,35.7)	53.6 (44.5,62.4)	<0.001

Abbreviations: 95% CI: 95% Confidence Interval, NCDs: Non-communicable diseases, OW/O: Overweight/Obesity; HT: Hypertension; DM2: Diabetes Mellitus 2; Dys: Dyslipidemia, GDA: Guide Daily Amount, WL: Warning Label

* GDA: n = 5,122, N expanded = 9,363,129; WL: n = 5,815, N expanded = 10,628,174

** GDA: n = 5,618, N expanded = 10,300,360; WL: n = 6,249, N expanded = 11,407,007

Most of participants who correctly classify the two products using both types of labeling were those with one NCD, which corresponds to participants with *OW/O*. 27.2% of participants identified product label 1 correctly using the GDA label (95%CI:24.5,30.1) and 39.5% (95%CI:(36.6,42.6) identified product label 2. However, almost doubled of the participants proportion classified product label 1 (51.9%, 95%CI:48.6,55.2) and product label 2 (56.4%, 95%CI:53.1,59.7) correctly using WL. By NCD type, double of the proportion of participants with *OW/O+HT+Dys* classified product label 1 correctly using WL (49.6%, 95%CI:40.3,58.9) compared to GDA label (21.9%, 95%CI:14.3,32.0) and nearly twofold the product label 2; 53.6% (95%CI:44.5,62.4) using WL vs. 27.3% (95%CI:20.3,35.7) using GDA for the same participants group.

The odds to classifying an unhealthy food product with the information contained in FoPL (Table 4) was higher using WL compared to GDA for both product labels. For product label 1 the probability was almost three times higher compared to participants who used GDA label, and the highest odds were found for participants with three or more diseases (OR = 2.88, 95%CI 2.0–4.1). For all participants, the likelihood of classifying product label 2 properly was two times higher when WL was used in comparison with those who used GDA; the highest odds were found for participants with two and three or more diseases (OR = 2.26, 95%CI:1.8–2.9 and 95%CI:1.7–3.0 respectively).

**Table 4:** Odds ratio of correct classification of two product labels using WL over GDA by number of diseases.

	Product label 1	Product label 2
	OR (95% CI)	OR (95% CI)
WL*Number of diseases ^ֆ^		
0	2.79*** (2.1,3.7)	2.18*** (1.7,2.8)
1	2.75*** (2.3,3.3)	2.01*** (1.7,2.4)
2	2.55*** (2.0,3.3)	2.26*** (1.8,2.9)
≥3	2.88*** (2.0,4.1)	2.26*** (1.7,3.0)

Abbreviations: 95% CI: 95% Confidence Interval, OR odds ratio, WL Warning Label.

*** P-value<0.001

^**†**^n = 14,880; N expanded = 25,650,088

^ֆ^Coefficients, confidence intervals and p value correspond to the interaction among WL and number of diseases.

Logistic model adjusted by sex, age, education level, region, locality, and socioeconomic level.

## Discussion

The results of the present study showed that adults, regardless their NCD status classified food products as unhealthy in a greater proportion using the WL compared to the GDA. The probability of correctly classifying a food product using the information contained on WL was twofold higher for all participants, being higher in those with three or more NCDs, which, according to NCD type corresponds mainly to participants with *OW/O+HT+Dys*. This is useful in the context of the epidemiological scenario of Mexico, as WL labeling was found to be more understandable than GDA and consequently could improve dietary decisions and promote the consumption of healthier diets.

The latest evaluations have shown that GDA labeling, as public policy, was poorly understood (24.6% correctly identified unhealthy foods) and consequently, not widely used (less than 10% used it for FAB selection [24]). Although our aim was not to evaluate labeling comprehension according to sociodemographic characteristics, other studies have reported that the use and comprehension of the former GDA labeling is lower in some population groups such as men with low educational and socioeconomic level and whose lives in rural regions of the country [29–31]; being less likely that Mexican adults with reported NCDs read the nutritional labeling compared to those with no reported NCD [29]. Its usage is related to difficulties in understanding qualitative labelling systems [32, 33], such as GDA, where Mexicans with self-reported NCDs, were not able to identified an unhealthy food product [24], this was consistent with the present study. In fact, the null or low use of nutrition labeling has been associated with a higher probability of developing NCDs compared to participants who use and read nutrition labeling [34], one of the possible factors that may contribute to the high prevalence of NCDs in Mexico. The usage of nutritional labeling in participants with and without NCDs differs across populations; in US and Malaysian population with NCDs reports a higher reading compared to population without NCD [35, 36]. However, in Korean and Thai population, as well as Mexican population, a lower reading of the nutritional labeling was observed in participants with NCDs [34, 37]. Some factors that may explain these differences are the socioeconomic and educational level [9, 10, 23, 24, 38], for example in Brazilian and Korean population whose label reading was higher in those with a high monthly income and educational level, regardless their presence or non-presence of NCDs [31, 37].

Our results obtained show that food product label 2 was identified as unhealthy in higher proportion compared to product label 1. The difference obtained in GDA participants is attributed to the false perception of healthiness of a food product when the GDA label contains many zeros [39], such as food product label 1, which contains zero in saturated and other fats. The difference found in the proportion using WL could be attributed to the greater number of warning symbols contained in the food product, which are three symbols in food product label 2 vs. two symbols in food product label 1; suggesting that participants classify an unhealthy food product more easily due to the number of WLs. In addition, our results show that participants with three or more NCDs classified food products correctly in a lower proportion compared to those who did not report NCDs or report a lower number of NCDs, these results could be attributed to different factors, such as age, education, socioeconomic level, and access to health services [24, 40, 41]. As we mentioned before, the socioeconomic level is associated with a lower understanding and reading of nutrition labeling, but also is associated with a reduced access to health services, therefore, scarce knowledge about the diseases they suffer [40, 42], factors that had been associated with a low management and control of the disease [43]. Another fact that could contribute to the low disease control is their food environment, which is unfavorable because in localities with low socioeconomic level the accessibility, availability and intake of UPPs is high [44–46]. Most of the UPPs available in Mexico exceed the amount of sodium allowed (68% of UPPs exceed the international benchmark targets) [47] leading to a higher incidence of HT and other NCDs [48, 49]. Some strategies implemented with the purpose of reduce their intake are taxation of sugar-sweetened beverages (SSBs) and junk food, increasing the availability and accessibility of healthy foods, improvement of healthy food standards in schools, regulation of food and beverage marketing to children, and implementation of a national FoPL [1, 21, 50]. Our results show that the WL is a simple-to-understand nutrition label for Mexican population, especially participants with NCDs.

Assessments of the effects on use, and other dietary outcomes of WL have not yet been reported. However, is expected that WL usage and understanding will be higher than the former GDA labeling, as it has been reported in countries that have implemented WL and experimental studies made in Latinos with low socioeconomic and education level [9, 17, 23, 51–54] and promotes healthier diets. Some reported benefits of WL is the reduction in the volume of purchases of SSB regardless the education level households [55], whose intake in Mexico is higher than other countries [56], and the purchase replacement of unhealthy food products with healthier ones [13] through the identification of products high in nutrients of concern [57, 58] leading to a reduction in their intake [53, 59]; and as a long-term result, a reduction in mortality caused by complications of these diseases [7]. It has been estimated that after five years of being implemented the WL in Mexico, caloric intake of UPPs will decrease by 36.8kcal/person/day on average, where most of them (23.2kcal) are from SSBs, having an impact on the reduction in the prevalence in obesity (it could be reduced by 15%) [60]. Given that the intake and purchase of UPPs in urban households with higher socioeconomic status is characterized by higher intakes of less healthy foods while households with lower socioeconomic status have higher purchases of less healthy beverages [61], the benefits of WL could be reflected in the entire population.

Our results suggests that the WL system could contribute to promote healthier diets, particularly those with three or more NCDs. Although differences were found in the socioeconomic level between the GDA and WL participants; the logistics models performed were adjusted for confounding variables included socioeconomic level. Another fact that is important to keep in mind is that the effect of WLs was possibly underestimated because the survey was conducted before the modification of NOM-051 and the label images of the food products shown contained different elements that were not included in NOM-051, such as the legends "this package provides" and "one serving provides".

The strengths of this paper include the objective evaluation of two FoPL, GDA labeling, and WL and the results obtained supply information at a national level since the data were obtained from a population-based survey designed to be representative of the Mexican population. Some limitations of the study is the usage of self-reported data of previous NCDs diagnosis, which can potentially underestimate real data, especially in indigenous and rural populations where prevalence has been found to be double that reported in national surveys [62]. Another limitation is the usage of BMI as the unique indicator of body fat since it does not differentiate between lean mass and fat mass; however, it is the most widely used anthropometric index for estimating overall body fat approved by the WHO [63]. Finally, that the survey was performed with images at the participants’ households, so this exercise would not represent the actual decision at the point of sale.

## Conclusions

The results found in this study showed that most Mexicans understand the nutritional information from WLs displayed in food and beverages products. The WL system helps consumers to classify unhealthy food products, regardless of their NCD status. However, participants with three or more NCDs classified unhealthy food products in a greater proportion. Classifying unhealthy products could result in a lower intake of critical nutrients and consequently in the consumption of healthier diets.
